# Probable vertical transmission of Alpha variant of concern (B.1.1.7) with evidence of SARS-CoV-2 infection in the syncytiotrophoblast, a case report

**DOI:** 10.3389/fmed.2022.1099408

**Published:** 2023-01-06

**Authors:** Hannah A. Bullock, Erika Fuchs, Roosecelis B. Martines, Mamie Lush, Brigid Bollweg, Alyssa Rutan, Amy Nelson, Mark Brisso, Albert Owusu-Ansah, Craig Sitzman, Laurie Ketterl, Tim Timmons, Patricia Lopez, Elizabeth Mitchell, Emily McCutchen, Jonathan Figliomeni, Peter Iwen, Timothy M. Uyeki, Sarah Reagan-Steiner, Matthew Donahue

**Affiliations:** ^1^Centers for Disease Control and Prevention, Atlanta, GA, United States; ^2^Nebraska Department of Health and Human Services, Lincoln, NE, United States; ^3^Bryan Health, Lincoln, NE, United States; ^4^Lincoln-Lancaster County Health Department, Lincoln, NE, United States; ^5^Nebraska Public Health Laboratory, Omaha, NE, United States; ^6^University of Nebraska Medical Center, Omaha, NE, United States

**Keywords:** SARS-CoV-2, vertical transmission, electron microscopy, histopathology, immunohistochemistry, case report

## Abstract

**Introduction:**

Definitive vertical transmission of severe acute respiratory syndrome coronavirus-2 (SARS-CoV-2) infection has been rarely reported. We present a case of a third trimester pregnancy with fetal distress necessitating cesarean section that demonstrated maternal, placental, and infant infection with the SARS-CoV-2 Alpha variant/B.1.1.7.

**Methods:**

CDC's Influenza SARS-CoV-2 Multiplex RT-PCR Assay was used to test for SARS-CoV-2 in a maternal NP swab, maternal plasma, infant NP swab, and formalin-fixed paraffin-embedded (FFPE) placental tissue specimens. Whole genome sequencing (WGS) was performed on maternal plasma, infant, and placental specimens to determine the SARS-CoV-2 genotype. Histopathological evaluation, SARS-CoV-2 immunohistochemistry testing (IHC), and electron microscopy (EM) analysis were performed on placenta, umbilical cord, and membrane FFPE blocks.

**Results:**

All specimens tested positive for SARS-CoV-2 by RT-PCR. WGS further revealed identical SARS-CoV-2 sequences from clade 20I/501Y.V1 (lineage Alpha/B.1.1.7) in maternal plasma, infant, and placental specimens. Histopathologic evaluation of the placenta showed histiocytic and neutrophilic intervillositis with fibrin deposition and trophoblast necrosis with positive SARS-CoV-2 immunostaining in the syncytiotrophoblast and electron microscopy evidence of coronavirus.

**Discussion:**

These findings suggest vertical transmission of SARS-CoV-2, supported by clinical course timing, identical SARS-CoV-2 genotypes from maternal, placental, and infant samples, and IHC and EM evidence of placental infection. However, determination of the timing or distinction between prepartum and peripartum SARS-CoV-2 transmission remains unclear.

## Introduction

Confirmed cases of vertical transmission of severe acute respiratory syndrome coronavirus-2 (SARS-CoV-2) appear to be rare (1), but case series and reports have described instances of possible transplacental SARS-CoV-2 transmission (2–6). However, timely ascertainment of laboratory evidence has been a barrier to confirmation (1, 2, 4). In this report, we present findings of SARS-CoV-2 infection in a symptomatic individual in the third trimester of pregnancy and infant delivered by cesarean section.

### Case description

A 27-year-old pregnant female (G2P1001 at 35 weeks, 5 days gestation) with an early childhood history of acute lymphocytic leukemia and no previous COVID-19 vaccination developed COVID-19 symptoms (Table 1), including chest pain and shortness of breath, and tested positive for SARS-CoV-2 by rapid antigen test on day 3 after onset of symptoms. Cough, severe dyspnea, and pleuritic chest pain developed on day 9. Computed tomography scan revealed a mild patchy ground-glass density in the lateral right middle lung lobe. A fetal non-stress test to evaluate heart rate and responsiveness was reassuring. Fever resolved on day 10 with improvement of dyspnea and chest pain on day 11, but cough and pleuritic chest pain continued. The mother expressed concern over reduced fetal movement and hospital evaluation was recommended. After a nonreactive fetal non-stress test with several fetal deceleration events, a cesarean section was performed the same day and was complicated by postpartum hemorrhage. The mother remained afebrile with improving cough and pleuritic chest pain and was discharged after 2 days on illness day 14.

**Table 1:** Case timeline: Maternal and neonatal symptom development, clinical care, and sample collection.

**Day**	**Patient**	**Event**	**Result of testing**
1	Mother	Onset of symptoms, including chest pain and shortness of breath	N/A
3	Mother	SARS-CoV-2 antigen test performed	Positive
9	Mother	Developed cough, severe dyspnea, and pleuritic chest pain; computed tomography (CT) scan with contrast performed, patient discharged	Chest CT scan negative for pulmonary embolism, mild patchy ground glass density was identified in the lateral right middle lobe
10	Mother	Fever resolved	N/A
11	Mother	Improved dyspnea and chest pain, cough and pleuritic chest pain continued, noted reduced fetal movement, cesarean delivery performed	Maternal plasma collected before cesarean delivery and placental tissue subsequently test positive for SARS-CoV-2 (see Results)
11	Neonate	In respiratory distress after birth, transported to the neonatal intensive care unit; chest X-ray performed	Right pneumothorax, bilateral ground glass densities, and other radiologic findings consistent with prematurity
12	Neonate	Respiratory status improved; SARS-CoV-2 nasal swab antigen test (collected 24 h after birth)	Negative
13	Neonate	SARS-CoV-2 nasal swab antigen test (collected 48 h after birth)	Positive
14	Mother	Discharged from hospital	N/A
14	Neonate	Reverse transcription polymerase chain reaction (RT-PCR) test on a nasopharyngeal (NP) swab (collected 72 h after birth)	Positive for SARS-CoV-2; negative for a panel of other respiratory viruses
16	Mother and Neonate	NP swabs collected from each individual	Swabs subsequently test positive for SARS-CoV-2 (see Results)
24	Neonate	Discharged from hospital	N/A

Apgar scores of the infant at birth were 7, 6, and 7, at 1, 5, and 10 min, respectively. Birthweight was 2,475 g and placental weight was 516 grams trimmed. The umbilical artery had a pH of 7.2. Meconium-stained amniotic fluid and double nuchal cord were present at delivery. The infant was in respiratory distress and brought to post-anesthesia care unit (PACU) for resuscitation. Suctioning revealed large amounts of meconium-stained fluid. The infant required continuous positive airway pressure (CPAP) with 5+cm H_2_O positive end expiratory pressure (PEEP) at 30% fraction of inspired oxygen (FiO_2_) at 4 min of life with an oxygen saturation (SpO_2_) of 58%. Due to increased work of breathing (SpO_2_ 70–80%), PEEP was increased to 6+cm H_2_0 and FiO_2_ was increased to 40% around 7 min of life. SpO_2_ improved to 88–90% and the infant was transported to the neonatal intensive care unit (NICU).

In the NICU, CPAP was switched to noninvasive neurally-adjusted ventilatory assist. Chest x-ray revealed a right pneumothorax and bilateral ground glass densities, and the infant was switched back to conventional CPAP at 6 h of life. The infant's respiratory status quickly improved and was weaned to room air on day 1 after birth. Nasal swabs collected 1 and 2 days after delivery tested negative and positive, respectively, by SARS-CoV-2 antigen test. A reverse transcription polymerase chain reaction (RT-PCR) test on a nasopharyngeal (NP) swab collected day 3 after birth was positive for SARS-CoV-2 and negative for a panel of other respiratory viruses. Delivery and neonatal care staff wore facemasks over N-95 respirators, or powered air-purifying respirators. The mother was masked throughout the delivery and did not have contact with the infant prior to NICU transfer.

The infant's pneumothorax resolved without chest tube placement. Routine hematologic and clinical chemistry testing on blood were normal for age. The infant was in airborne isolation for 10 days and was discharged at 13 days old feeding appropriately and gaining weight. Subsequently, identical SARS-CoV-2 Alpha/B.1.1.7 genotypes were identified in specimens from mother, infant, and placenta. Placenta histology was consistent with previous reports of placental SARS-CoV-2 infection (1, 4, 7, 8). Additionally, immunostaining for SARS-CoV-2 and electron microscopy (EM) analysis of the placenta found evidence of coronavirus in the syncytiotrophoblast.

## Materials and methods

This study was reviewed by the Centers for Disease Control and Prevention (CDC) and was conducted consistent with applicable federal law and CDC policy.^§^ Written informed consent for this work was obtained from the mother for herself and from both parents for the infant. Nebraska Public Health Laboratory (NPHL) received four specimens for analysis: maternal NP swab collected 5 days post-delivery, maternal plasma collected approximately 30 min before delivery, infant NP swab collected 5 days post-birth, and formalin-fixed, paraffin-embedded (FFPE) placental tissue. Nucleic acid isolation was performed using the MagMAX™ Viral/Pathogen II Nucleic Acid Isolation Kit and KingFisher Flex System (Thermo Fisher Scientific) for NP swabs, QIAamp Viral RNA Mini Kit (QIAGEN) and QIAcube Connect for serum, and RNeasy FFPE Kit (QIAGEN) for RNA isolation from FFPE tissue. All specimens underwent RT-PCR testing using CDC's Influenza SARS-CoV-2 Multiplex Assay on the Applied Biosystems™ 7500 Fast Dx RT-PCR Instrument (Thermo Fisher Scientific).

Whole genome sequencing (WGS) was performed using the Clear Dx™ WGS SARS-CoV-2 kit and Clear Labs Dx™ System (Clear Labs), composed of a GridION (Oxford Nanopore) and Hamilton STAR liquid handler. The Clear View WGS application (https://wgs.app.clearlabs.com) was used for assembly and sequencing coverage analysis. RNA mutation identification used Nextclade version v0.14.2 (https://clades.nextstrain.org). Sample lineages were assigned using the Pangolin COVID-19 Lineage Assigner (https://pangolin.cog.uk.io). Clonality between samples was determined *via* single nucleotide polymorphism (SNP) analysis using SNP-sites/2.5 with default parameters (https://github.com/sanger-pathogens/snp-sites).

The CDC Infectious Diseases Pathology Branch performed histopathological evaluation and SARS-CoV-2 immunohistochemical (IHC) and EM analysis of placenta, umbilical cord, and membrane FFPE blocks. Placenta, umbilical cord, and membrane tissues had been received fresh by the pathologist in Nebraska and fixed in 10% neutral buffered formalin and embedded in paraffin to create FFPE blocks. IHC assays used a rabbit polyclonal antibody raised against SARS-CoV-2 nucleocapsid (GenTex-GTX635686) at 1:100 dilution and a mouse monoclonal SARS-CoV-2 spike (GeneTex-GTX632604) at dilution 1:250 with a Mach 4 Universal AP Polymer Kit (Biocare Medical) with Permanent Red Chromogen (Cell Marque). Slides were pretreated with heat-induced epitope retrieval with citrate-based buffer (Biocare Medical). Negative controls, run in parallel, used normal rabbit, or mouse serum in place of the primary antibody. FFPE samples for EM were processed as described in Martines et al. (9).

## Results

RT-PCR detected SARS-CoV-2 RNA in the maternal NP swab, maternal plasma, infant NP swab, and FFPE placental tissue specimens. Cycle threshold (Ct) values, identified from graphical outputs, were 29.8, 25.7, 16.5, and 14.4 for the maternal NP swab, maternal plasma, infant NP swab, and placental tissue, respectively. The test's Ct cutoff value was 40. WGS performed on the maternal plasma, infant, and placental specimens revealed all SARS-CoV-2 sequences belonged to clade 20I/501Y.V1 (lineage Alpha/B.1.1.7) and were 100% identical along the 29902-bps analyzed with no SNP differences. Sequencing was not performed on the maternal NP swab due its high Ct value (29.8).

**Figure 1:**
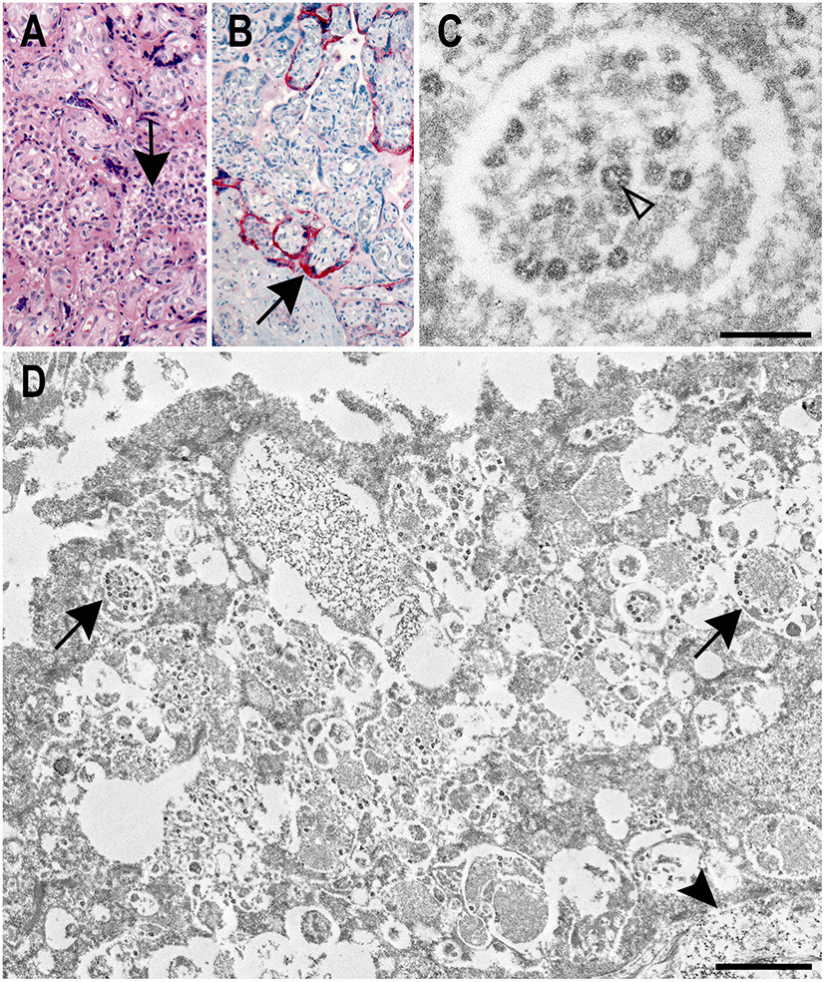
Correlative histology, immunohistochemistry, and ultrastructural findings in a SARS-CoV-2 positive placenta. **(A)** Placenta showing histiocytic intervillositis (arrow) with villous trophoblast necrosis and fibrin (H&E, original magnification 20X). **(B)** Immunostaining of SARS-CoV-2 nucleocapsid protein (arrow) in the syncytiotrophoblast of the placenta (IHC, original magnification 20X). **(C)** Electron microscopy image displaying a membrane bound accumulation of coronavirus particles in the syncytiotrophoblast. Cross sections through the viral nucleocapsid visible (open arrowhead). Scale bar: 200 nm. **(D)** Vacuolar, membrane bound accumulations of coronavirus particles (arrows) in the syncytiotrophoblast of the placenta as seen by electron microscopy. A cytotrophoblast (arrowhead) is visible below the syncytiotrophoblast. Scale bar: 1 μm.

Placenta histology displayed prominent histiocytic and neutrophilic intervillositis with fibrin deposition and trophoblast necrosis (Figure 1A). Umbilical cord and membranes showed no significant histopathologic findings. IHC assays using antibodies against SARS-CoV-2 nucleocapsid and spike proteins showed extensive staining in villous syncytiotrophoblast and intervillous inflammatory cells (Figure 1B).

EM revealed particles morphologically consistent with coronavirus in the syncytiotrophoblast (Figures 1C,D). Areas for EM analysis were identified from IHCs showing positive staining for the SARS-CoV-2 spike protein (Figure 1B). Membrane-bound viral particles displayed visible cross-sections through the viral nucleocapsid and were on average 66 nm in diameter (range 56–81 nm; Figure 1C). The size of these particles was smaller than is typical of a coronavirus due to the use of FFPE tissues. Spikes were not readily apparent on the viral particles; viral spikes are typically not visible on intracellular coronavirus viral particles. Though the ultrastructure of the specimen was diminished due to the use of FFPE tissues, margins of the syncytiotrophoblast and adjacent cytotrophoblast were discernable as were vacuolar accumulations of viral particles within the syncytiotrophoblast cytoplasm (Figure 1D).

## Discussion

Though rare, vertical transmission of SARS-CoV-2 has been reported (1, 3, 5, 6, 8, 10, 11), however, conclusive cases remain elusive. This report presents evidence indicating probable vertical transmission of SARS-CoV-2, supported by clinical course timing, lack of contact between mother and infant postpartum, and identical genotypes of SARS-CoV-2 identified from both patients' specimens, including maternal plasma before cesarean delivery. However, determination of the timing or distinction between prepartum and peripartum SARS-CoV-2 transmission remains unclear due partially to specimen availability.

Previous SARS-CoV-2 studies indicate placental infection is necessary before fetal infection and *in utero* infection requires that virus crosses the maternal-placental interface to access fetal vessels (12). We demonstrated placental SARS-CoV-2 infection by IHC, as recommended by Roberts et al. (7), showing localization of SARS-CoV-2 nucleocapsid and spike proteins in the syncytiotrophoblast, consistent with published reports (13, 14). Positive staining has been reported in Hofbauer cells and villous capillary endothelial cells (14), but was not observed in this case. EM further verified coronavirus infection of the syncytiotrophoblast. The presence of numerous membrane bound collections of coronavirus particles within the syncytiotrophoblast suggest the virus is replicating in this cell type. As the syncytiotrophoblast is the initial defense against pathogens attempting to cross the placental barrier and is in direct contact with maternal blood, the virus observed in this cell type is further evidence of maternal viremia. Since coronavirus was not identified by EM in other areas of the placenta, whether the virus entered fetoplacental circulation cannot be determined. Even now, accurate reports of coronavirus particles in the placenta have been rare (15, 16) with several articles misidentifying common subcellular structures as coronavirus in the placenta as well as other tissues (17).

Placental histopathologic evaluation further revealed chronic histiocytic intervillositis, trophoblastic necrosis, and increased perivillous fibrin deposition, all described as hallmarks of SARS-CoV-2 placental infection (7, 8, 11). However, the intervillositis observed may be an incidental finding or SARS-CoV-2 infection may have exacerbated existing intervillositis. The potential causes of intervillositis are varied and not well understood, though the condition is hypothesized to result from excessive maternal inflammation toward the placenta, possibly due to maternal infection in some cases (18, 19). Intervillositis has been reported in other placentas from SARS-CoV-2 positive individuals (13, 20, 21), but has also been observed with other infectious diseases including dengue (22) and cytomegalovirus (23). Larger cohort studies are necessary to determine any correlation between SARS-CoV-2 infection and adverse pregnancy outcomes including intervillositis.

Together, the data presented enable this case to meet the definition for probable SARS-CoV-2 vertical transmission (12) and serve as an example of the importance of comprehensive sample collection. Detection of SARS-CoV-2 RNA in maternal plasma before cesarean delivery suggests viremia, while RT-PCR, IHC, and EM results indicated the presence of SARS-CoV-2 in placental tissues. SARS-CoV-2 infection in the neonate was confirmed on days 2 and 3 after delivery, by antigen test and RT-PCR, respectively. Unfortunately, a specimen from the infant on the day of delivery was not available for RT-PCR testing. Meconium-stained amniotic fluid was present at delivery and a large amount of meconium-stained fluid was suctioned from the infant's lungs. However, for meconium aspiration to be the route of transmission, SARS-CoV-2 would need to be present in the meconium either prior to birth or present in amniotic fluid the infant aspirated along with meconium during delivery. While amniotic fluid and meconium were not available for testing in this case, other studies have noted a higher viral load in placental tissue than in amniotic fluid (20) and amniotic fluid from 43 SARS-CoV-2 positive pregnant mothers tested negative for SARS-CoV-2 by RT-PCR (24). Additionally, a systematic review by Allotey et al., found infection of amniotic fluid or placental tissue with SARS-CoV-2 did not necessarily correlate with fetal infection (1). Still, the lack of a specimen from the infant on the day of delivery or an amniotic fluid or meconium specimen limit definitive conclusions about the timing of vertical transmission.

Thankfully, this case had a positive outcome with the full recovery of both mother and infant. At the time of this delivery, maternal SARS-CoV-2 vaccination was an individual decision. CDC, the American College of Obstetricians and Gynecologists, and the Society for Maternal-Fetal Medicine now strongly recommend vaccination to prevent SARS-CoV-2 infection before or during pregnancy (25).

## Ethics statement

This activity was reviewed by CDC and was conducted consistent with applicable federal law and CDC policy.^§^ Written informed consent was obtained. ^§^See e.g., 45 C.F.R. part 46, 21 C.F.R. part 56; 42 U.S.C. §241(d); 5 U.S.C. §552a; 44 U.S.C. §3501 et seq. Written informed consent to participate in this study was provided by the participants' legal guardian/next of kin. Written informed consent was obtained from the individual(s), and minor(s)' legal guardian/next of kin, for the publication of any potentially identifiable images or data included in this article.

## Author contributions

HB and EF contributed equally to conceptualization, design, and drafting of the manuscript. HB, EF, RM, TU, SR-S, and MD contributed to study design, data review, and manuscript revision. AR, AN, and MB participated in patient treatment. AO-A and LK participated in neonate care. Initial investigation and gathering of case information was performed by ML, TT, PL, and MD. MD, PI, CS, and JF participated in specimen and testing coordination. CS participated in specimen collection. Laboratory testing and review of results was performed by HB, BB, RM, EMi, and EMc. ML, HB, MD, EMi, EMc, RM, BB, and TU contributed to the interpretation of laboratory results. All authors reviewed and approved of the final manuscript.
